# An Alternative Approach for Treating Female Underactive Bladders with Chronic Urine Retention: A Pilot Study on Combined Transvaginal Ultrasound-Guided Botulinum Toxin A External Sphincter Injection and Transurethral Incision of the Bladder Neck

**DOI:** 10.3390/toxins16100441

**Published:** 2024-10-14

**Authors:** Wei-Chun Huang, Cheng-Yen Tsai, Eric Chieh-Lung Chou

**Affiliations:** China Medical University Hospital, Taichung City 404, Taiwan; chsh010795@gmail.com

**Keywords:** underactive bladder, chronic urine retention, botulinum toxin A, transvaginal ultrasound guidance, external sphincter injection, transurethral incision of the bladder neck

## Abstract

**Background:** Treating an underactive bladder (UAB) is challenging. Previously, we introduced a more precise method of transvaginal ultrasound-guided botulinum toxin A (BoNT-A) injection into the external urethral sphincter as a treatment option for patients with UABs. Although many patients experience good results, those with an UAB and excessive residual urine still require catheterization. Therefore, we developed a new method that combines transvaginal ultrasound-guided BoNT-A injection with a transurethral bladder neck incision. **Methods:** A prospective study was conducted on 16 patients who experienced symptoms of UAB and chronic urine retention. The treatment consisted of a combination of transvaginal ultrasound-guided BoNT-A injection and a transurethral incision of the bladder neck (TUI-BN). The primary objective was to assess the efficacy of this combined treatment in improving symptoms in women with UABs. **Results:** Our study demonstrated significant improvements after treatment, including increased voiding volume, decreased post-void residual (PVR) urine, and improved voiding efficiency. The frequency of clean intermittent catheterization (CIC) decreased at 1 and 3 months post-surgery, along with improvements in the AUA symptoms score and the Patient Perception of Bladder Condition (PPBC) score. **Conclusions:** Our study showed significant improvements in the surgical treatment of UABs using a combination of transvaginal ultrasound-guided BoNT-A and TUI-BN.

## 1. Introduction

An underactive bladder (UAB) is defined by the International Continence Society as a symptom complex characterized by a slow urinary stream, hesitancy, and straining to void, with or without a feeling of incomplete bladder emptying and sometimes with storage symptoms [1]. It is a lower urinary tract symptom (LUTS) occurring in up to 45% of females [1]. Clinically, a patient with a UAB primarily presents with symptoms during the voiding phase, including decreased urine flow rate, dribbling, hesitancy, and a sensation of incomplete bladder emptying. In recent years, there has been an increasing interest among physicians owing to its significant impact on patients’ quality of life and the complexity of its management. Despite this, a definitive treatment that is capable of fully curing UAB and substantially improving patients’ quality of life remains elusive.

An untreated UAB can lead to acute and chronic urine retention, hydronephrosis, urinary tract infections, and even renal failure, posing a substantial threat to patient well-being [2,3]. Therefore, current UAB management primarily focuses on reducing residual urine and protecting the urinary tract from further damage [4,5]. According to the 2023 European Association of Urology (EAU) guidelines, existing therapies include conservative treatment, pharmacotherapy, electrical stimulation, and surgery [6,7]. Among these treatment methods, botulinum toxin A (BoNT-A) injections into the urethral sphincter are often chosen [8,9,10]. However, the duration of the effect of this treatment is short, lasting only 3–9 months, necessitating repeated treatments to maintain efficacy [11,12]. Another surgical option is a transurethral incision of the bladder neck (TUI-BN); however, satisfactory outcomes have been reported in only 48.8% of cases [13]. This highlights the need for more effective treatments that can offer better relief and improve patient outcomes, especially for those with more severe symptoms. The limitations of current therapies underscore the importance of continued research and innovation in UAB treatment.

This study aimed to identify treatments for patients with UABs and chronic urine retention to achieve better outcomes while minimizing complications. Specifically, we investigated a novel approach that combined transvaginal ultrasound-guided BoNT-A and injection with TUI-BN.

## 2. Results

The patient characteristics are presented in Table 1. The average age of the patients was 66.1 ± 11.5 years. The mean PVR volume was 358.1 ± 192.3 mL, and the VE was approximately 24.6 ± 20.5%. Preoperatively, the VUDS was conducted, and all patients’ findings revealed detrusor underactivity.

Table 2 presents the outcomes, comparing the preoperative and postoperative profiles. The PVR urine volume, VE, and frequency of CIC all showed significant improvements at both 1 month and 3 months after surgery compared to preoperative values.

Regarding the questionnaires, both the AUA symptoms score and PPBC scores showed statistically significant improvements at both 1 and 3 months postoperatively, whereas the AUA symptoms score—QoL demonstrated a significant enhancement 3 months after surgery. Our study indicated that 88% of the patients experienced improved voiding by more than 50% at both 1 and 3 months after surgery.

The subgroup analysis of the AUA symptoms score revealed that voiding symptoms improved at both 1 and 3 months after surgery, whereas storage symptoms improved only at 3 months postoperatively. None of the 16 patients experienced major adverse events or complications following the surgery. One patient had mild stress urinary incontinence (SUI) preoperatively, which progressed to more severe SUI during the outpatient department follow-up 3 months after surgery. Conservative treatment was attempted for 3 months, but the outcomes were unsatisfactory. We then performed retropubic sling surgery, which resulted in good voiding outcomes without obvious incontinence. There were no reports of genitourinary tract injury, major bleeding, or vesicovaginal fistulae in any patient.

## 3. Discussion

Currently, there is no definitive treatment for a UAB. According to the EAU guidelines, CIC is the recommended standard of care for patients who cannot effectively empty their bladders [2,6,14]. Previous reviews have highlighted conservative methods, pharmacotherapy, and surgical interventions; however, all have shown limited effectiveness in improving voiding function [2,15,16].

According to the guidelines of the EAU, surgical intervention involving BoNT-A intersphincteric injections into the external urethral sphincter can improve voiding by reducing the outlet resistance and suppressing the guarding reflex. BoNT-A inhibits neurotransmitter release at neuromuscular junctions, leading to muscle paralysis [17]. However, a drawback of this method is its relatively short duration of effectiveness, typically between 3 and 9 months, necessitating repeated injections to maintain efficacy [11,12]. Furthermore, several studies have compared the outcomes of different doses of BoNT-A injected into the external urethral sphincter. Previous research has suggested that the recommended doses for urethral sphincter injections range from 50 to 200 units, depending on the underlying condition [18,19]. Nevertheless, determining the optimal dose of BoNT-A injections into the external sphincter remains a persistent and unresolved challenge.

Reviewing previous research, a 100-unit dose of BoNT-A has been widely recommended and utilized to reduce urethral resistance and facilitate effective voiding [20,21,22,23]. Kuo et al. reported that injecting 100 units of BoNT-A into the urethral sphincter of patients with detrusor underactivity resulted in an average efficacy duration of 8.4 months, with 68% of patients achieving excellent outcomes [12]. In contrast, Jiang et al. found a lower overall success rate of 43.5% for BoNT-A 100 U injections into the external sphincter in patients with non-neurogenic voiding dysfunction [24]. Tsai et al. introduced a precise method involving the ultrasound-guided injection of 200 units of BoNT-A into the external sphincter in patients with UABs. Postoperative improvements were observed in AUA symptoms score, PPBC, and QoL on AUA symptoms score. Additionally, patients experienced significantly reduced PVR volumes and frequencies of CIC. Despite the effectiveness of this inspiring method, some patients still suffer from excessive urine retention and require CIC. This has sparked our interest in combining this surgical technique with other procedures to determine whether the surgical outcomes can significantly reduce the need for catheterization or improve CIC outcomes for patients.

Successful surgical outcomes of TUI-BN for female patients with detrusor underactivity and urinary retention have been previously documented [13,24,25,26,27,28]. In video urodynamic studies, Lee et al. observed that approximately 20% of women diagnosed with detrusor underactivity and urinary retention demonstrated inadequate bladder neck opening when attempting to void with abdominal straining [13]. In their study, they conducted the TUI-BN at the 5 o’clock and 7 o’clock positions. The postoperative results showed that 48.8% of the patients had satisfactory outcomes after a single TUI-BN procedure, and 60.9% of the patients were able to void spontaneously with abdominal straining, eliminating the need for catheterization. Regarding adverse events, 6.1% of patients experienced SUI, and 2.4% had vesicovaginal fistula [13].

The mechanisms underlying the efficacy of TUI-BN in the treatment of UABs remain unclear and controversial. One proposed mechanism suggests that the bladder neck is innervated by the sympathetic adrenergic nerves, which play a crucial role in the guarding reflex during the micturition cycle [29]. Previous studies have indicated that terminal nerves are densely distributed at the 4 o’clock and 8 o’clock positions in the bladder neck [30]. Additionally, urethral sphincter contraction inhibits bladder contraction [31]. Damage to the bladder neck may disrupt this reflex pathway and attenuate sympathetic overactivity, potentially reducing the detrusor muscle contractions in the spinal cord. Patients with idiopathic detrusor underactivity might experience improved voiding owing to this effect, suggesting that TUI-BN could trigger a reflex facilitating micturition [27,32,33].

Therefore, we propose treating patients with detrusor underactivity using a combination of transvaginal ultrasound-guided BoNT-A injection and TUI-BN. In the current study, the surgical approach aimed to achieve relaxation of the external urethral sphincter through BoNT-A injections and to alleviate bladder outlet pressure via TUI-BN. Our purpose in combining these two treatments was to effectively reduce the pressure at the bladder outlet, allowing female patients to void using abdominal pressure, rather than focusing on enhancing the bladder contractility. It should be noted that the effects of botulinum toxin may take 1–2 weeks to manifest after surgery; therefore, effectiveness should not be expected immediately after treatment [11]. Patients may still need to perform self-catheterization initially; therefore, it is essential for them to learn this procedure before undergoing surgery. If self-catheterization is not mastered, it is recommended to leave the catheter in place for 2–3 weeks before attempting spontaneous urination. Alternatively, the use of a suprapubic catheter is a viable option for bladder rehabilitation.

In our study, 88% of the patients achieved voiding capacity of more than 50% 1 month after surgery, and this efficacy was maintained at 3 months postoperatively. The PVR urine volume decreased significantly from 358.1 ± 192.3 to 118.3 ± 75.0 (*p* ≤ 0.001 *) at 1 month and to 76.7 ± 73.8 (*p* ≤ 0.001 *) at 3 months after surgery. Additionally, the average number of CIC sessions per day improved from 3.4 ± 2.4 before surgery to 1.3 ± 1.1 (*p* = 0.002 *) at 1 month and 0.5 ± 1.1 (*p* = 0.002 *) at 3 months post-surgery. Only four patients continued to require self-catheterization, while the remaining patients with detrusor underactivity were able to void spontaneously without CIC. These results are remarkable compared to previous findings. Furthermore, improvements were observed in questionnaire scores: the AUA symptoms score decreased from 24.6 ± 5.3 initially to 12.3 ± 6.9 (*p* = 0.001 *) at 1 month and to 8.0 ± 5.9 (*p* ≤ 0.001 *) at 3 months post-surgery; the PPBC score decreased from 4.4 ± 1.0 to 2.1 ± 1.2 (*p* = 0.001 *) at 1 month and to 0.7 ± 0.9 (*p* ≤ 0.001 *) at 3 months post-surgery. The AUA symptoms score—QoL also showed improvement 3 months after surgery, decreasing from 4.9 ± 1.1 to 1.3 ± 0.9 (*p* = 0.001 *).

We also analyzed the AUA symptoms score subgroup, focusing on storage and voiding symptoms. Storage symptoms improved from 8.7 ± 2.7 to 3.8 ± 3.1 at 3 months postoperatively, whereas voiding symptoms improved from 15.9 ± 3.9 to 2.8 ± 1.1 (*p* ≤ 0.001 *) at 1 month and to 4.2 ± 3.7 (*p* ≤ 0.001 *) at 3 months after surgery. These findings are consistent with our hypothesis that our approach did not primarily enhance bladder contractility in the short term but rather reduced urethral resistance and facilitated spontaneous voiding through abdominal straining.

No instances of bleeding, hematoma, or vesicovaginal fistula formation occurred after surgery. Moreover, most patients did not have any significant issues with urinary incontinence, while most of them experienced only very mild SUI, which can be greatly improved through pelvic floor muscle training, and no special medication is required for treatment. However, in our study, one of the patients had relatively obvious SUI before surgery. The patient was a 39-year-old woman who worked as a nanny and needed to commute frequently to care for infants. The patient was able to void with abdominal straining at 1 month postoperatively. Although she was very satisfied and free from urine retention, she complained of more noticeable urinary incontinence and needed 5–6 pads per day. In this particular case, after 3 months of conservative treatment with unsatisfactory results, we proceeded with retropubic sling surgery [8]. Sling surgery was performed in the mid-urethra. Under normal conditions, this does not significantly increase urethral resistance. However, when the intra-abdominal pressure increases, such as during coughing, sneezing, or lifting heavy objects, it can instantly elevate the outlet resistance, thereby alleviating the symptoms of urinary incontinence. This implies that even if patients with exacerbated SUI following our treatment for a UAB undergo any sling surgery, it will not negate the benefits of our previous treatment efforts. This is because transvaginal ultrasound-guided BoNT-A urethral injection relaxes the external sphincter, whereas TUI-BN reduces the resistance from the internal sphincter. If patients have noticeable SUI before surgery, they should be informed beforehand that urinary incontinence may worsen after surgery.

Our study has several limitations that must be acknowledged. First, the study was conducted at a single center with a small sample size, which inherently restricts the generalizability of the findings. The limited sample size may not adequately represent the broader population, potentially biasing the results. Second, the follow-up period was relatively short, which may not have provided a comprehensive overview of the long-term outcomes of the surgical interventions. Longer follow-up periods are necessary to fully understand the long-term effects and potential complications of the treatment.

Additionally, our data collection relied on face-to-face questionnaires during outpatient follow-up. This method has the potential to introduce bias, as patients may overestimate their improvements owing to the presence of healthcare providers or recall bias. Moreover, most patients in this study had complex and severe voiding dysfunctions that were resistant to conventional treatments, which could have led to a selection bias. This variability may have impacted the study outcomes, as these patients responded differently to surgical intervention than those with less severe conditions. Whether patients with mild UAB would benefit from this surgical approach remains unclear, highlighting the need for further investigations.

Finally, this was a single-arm study and did not include a placebo or control group. This design makes it challenging to determine whether the combined treatment is superior to BoNT-A 100U or TUI-BN alone. The absence of a comparative group restricts our ability to establish the relative efficacy of the combined treatment conclusively. Future studies should address this aspect by including a placebo group or comparing combined treatment with each intervention separately.

## 4. Conclusions

Our study demonstrated significant improvements in the surgical treatment of UABs using a combination of transvaginal ultrasound-guided BoNT-A injection and TUI-BN. This approach resulted in a notable decrease in PVR urine, improved VE, and nearly eliminated the need for CIC 3 months after the intervention. Additionally, patients showed improved AUA symptoms score, AUA symptoms score—QoL, and PPBC scores at 3 months post-surgery. No major complications were observed after surgery, highlighting the safety of this combined surgical method. These results suggest that the dual approach addresses both the mechanical and functional aspects of bladder dysfunction and provides significant benefits to patients. Although promising, these data must be strengthened by randomized blind-controlled clinical trials comparing the double treatment with each single treatment. The combination of transvaginal ultrasound-guided BoNT-A injection and TUI-BN appears to offer substantial improvements in both objective bladder function measures and patients’ subjective experience. Future studies should further explore this treatment to confirm its long-term efficacy and safety in larger cohorts.

## 5. Material and Methods

This study was conducted as a prospective study of 16 women with UAB symptoms and chronic urine retention (Table S1). These patients underwent transvaginal ultrasound-guided BoNT-A injection combined with TUI-BN as a surgical intervention for UABs at China Medical University Hospital between January 2023 and December 2023. All patients had experienced urine retention for at least 1 year and were dissatisfied with previous conventional treatments, including alpha-blocker and bethanechol therapies. All the patients exhibited either detrusor underactivity or an acontractile detrusor. Patients with pelvic organ prolapse, severe systemic diseases (such as uncontrolled cancer, coronary artery disease, or end-stage renal disease), and an Eastern Cooperative Oncology Group Performance Status >3 were not considered candidates for surgery and excluded from the study. Additionally, patients with a documented history of previous anti-incontinence surgical interventions or spinal cord injury were excluded.

Our study received approval from the Institutional Review Board and the Ethics Committee of China Medical University Hospital, Taichung, Taiwan (DMR-94-IRB-083).

Informed consent was obtained from all the participants. All patients underwent a video urodynamic study (VUDS) in accordance with the recommendations of the International Continence Society. Age, body mass index, underlying diabetes mellitus, recurrent urinary tract infections, and surgical history were also recorded. Voiding diaries were maintained to analyze the patients’ preoperative and postoperative voiding conditions. We also assessed the patients’ voiding improvement using questionnaires, including the AUA symptoms score, AUA symptoms score—QoL index, and PPBC, through direct interviews conducted by the doctor before and after surgery. There were no missing values in the baseline characteristics, VUDS, or questionnaires. The primary objective of this study was to evaluate the efficacy of the combination of transvaginal ultrasound-guided BoNT-A injection and TUI-BN.

### 5.1. Video Urodynamics Study

A C-arm fluoroscope and a multichannel urodynamic system were used [34]. The VUDS was repeated at least twice to ensure a reproducible pressure flow trace. Data collected included the first sensation of filling, cystometric bladder capacity, maximum flow rate (Qmax), detrusor pressure at Qmax (PdetQmax), PVR—urine amount, sphincter electromyography activity, voided volume, bladder contractility index (BCI, defined as BCI = PdetQmax + 5 Qmax), and voiding efficiency (VE, defined as voided volume/bladder capacity × 100%) [35]. During the VUDS, voiding cystourethrography was performed using a C-arm fluoroscope positioned at a 45-degree angle from the buttocks to optimize the visualization of the bladder neck, urethral sphincter, and distal urethra by elongating the urethra.

### 5.2. A Novel Treatment: Surgical Treatment with a Combination of Transvaginal Ultrasound-Guided BoNT-A Injection and TUI-BN

Currently, according to the EAU guidelines, surgical intervention with BoNT-A urethral sphincter injections into the external urethral sphincter and TUI-BN has been approved for the treatment of UAB, although it has shown limited improvement [6,36].

Our previous study in 2023 proposed transvaginal ultrasound-guided BoNT-A injection as a more precise method for targeting the external urethral sphincter, which yielded excellent results [37]. This approach is considered more accurate, because the external urethral sphincter in women is very thin (approximately 2 mm thick) and surrounded by connective tissue [38,39]. Using ultrasound guidance, we can dynamically adjust the injection site and increase the BoNT-A dosage accuracy to the external urethral sphincter.

This study evaluated the efficacy of the described combination treatment. Additionally, these patients were monitored for postoperative adverse events using the Clavien–Dindo classification system.

**Figure 1:**
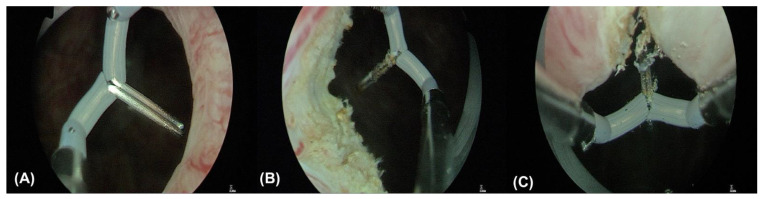
Transurethral incision of bladder neck (**A**–**C**). Incisions are made at the 4, 8, and 12 o’clock positions of the bladder neck, extending deeply into the circular fibers without perforation. The distal part of the incisions is carefully made to avoid injury to the external sphincter.

**Figure 2:**
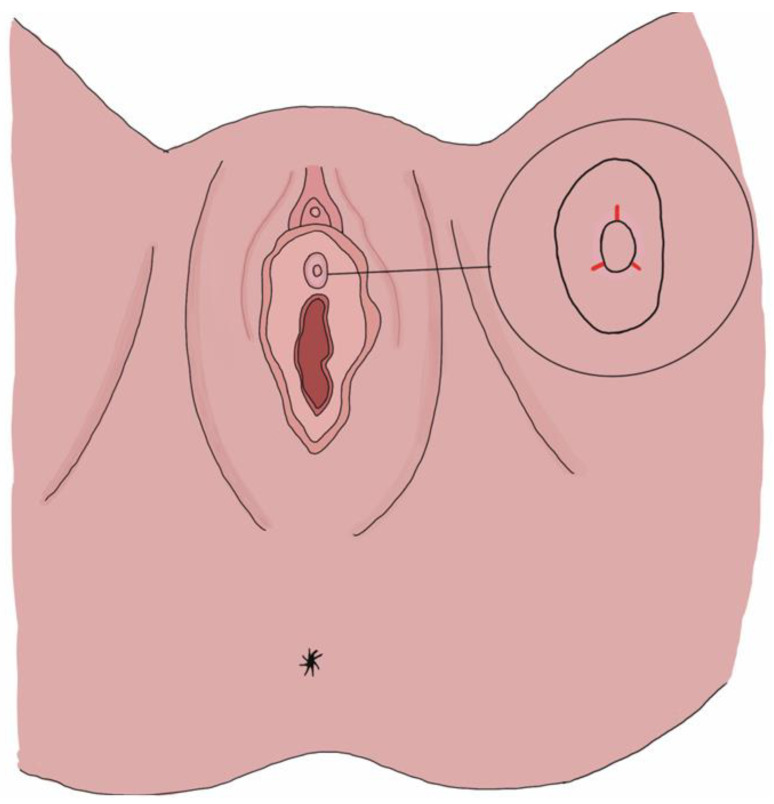
Schematic diagram of the TUI-BN site. We make three incisions at the 4, 8, and 12 o’clock positions on the bladder neck.

**Figure 3:**
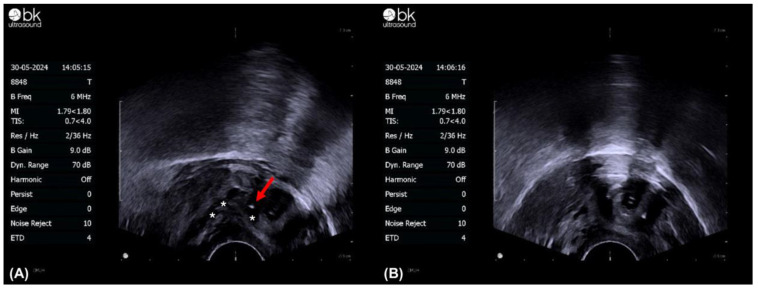
Transvaginal ultrasound-guided botulinum toxin A external urethral injection. (**A**,**B**). The external urethral sphincter’s (dark area, marked as *) precise location can be identified through transvaginal ultrasound; injection of BoNT-A; the tip of the needle is marked by a red arrow.

The patient was placed in the lithotomy position under laryngeal mask airway anesthesia. The external genitalia were sterilized using povidone-iodine. We first performed a 70-degree cystoscopy to check for the presence of vesical stones, bladder tumors, or other lesions. An Olympus 26 F transurethral resectoscope with a needle-type electrode was used, utilizing a monopolar energy of 135 W for cutting and 80 W for coagulation. After fully distending the bladder, three incisions were made at the 4, 8, and 12 o’clock positions on the bladder neck (Figure 1 and Figure 2). Using diathermy, incisions were made deep into the circular fibers of the bladder neck, extending distally by approximately 1.5 cm. We identified the location of the external sphincter to avoid damage. Following the TUI-BN procedure, we proceeded with the transvaginal ultrasound-guided BoNT-A injection according to the surgical steps described by Tsai et al. (Figure 3). A single 16 Fr. Foley catheter was inserted into the urethra, and 150 mL of saline solution was infused to enhance the visibility of the external sphincter. We then administered 100 units of BoNT-A into the external sphincter under ultrasound guidance, targeting the 2–4 o’clock and 8–10 o’clock positions, with 1 cc per site. Using transvaginal ultrasonography, we precisely injected diluted BoNT-A (100 units in 4 cc saline) into various parts of the external sphincter.

The patient retained the Foley catheter for one day post-surgery. We educated the patients on the number of CICs required based on the catheterization amount.

During outpatient follow-up, we re-evaluated the parameters using the following questionnaires: AUA symptoms score, QoL on the AUA symptoms score, and PPBC at 1 month and 3 months after surgery.

### 5.3. Data Analysis

Demographic and VUDS parameters were recorded as either categorical or continuous variables. Additionally, questionnaire assessment scores, PVR urine volume, VE, and CIC data before and after the combination of transvaginal ultrasound-guided BoNT-A injection and TUI-BN were evaluated. Statistical analyses were performed using SPSS for Windows (version 22.0). The paired Wilcoxon rank-sum test was used to determine the statistical differences for continuous variables, whereas categorical variables were compared using Fisher’s exact test. Statistical significance was set at 0.05. Postoperative complications following the surgery were documented in this study.

## Figures and Tables

**Table 1:** Patient characteristics.

	Patient (*n* = 16)
Age (years)	66.1 ± 11.5
BMI (kg/m^2^)	22.3 ± 3.1
Diabetes Mellitus	3
Recurrent urinary tract infection	0
Neurogenic disease	1
Abdominal operation history	2
Voiding volume (VV) (mL)	127.8 ± 172.0
PVR (mL)	358.1 ± 192.3
VE(%)	24.6 ± 20.5
Cystometry	
First sensation of filling (FSF) (mL)	155.8 ± 94.3
Cystometric bladder capacity (CBC) (mL)	442.5 ± 181.6
Pdet (cmH_2_O)	24.8 ± 14.2
Pabd (cmH_2_O)	11.6 ± 12.9
Qmax	3.3 ± 3.6
Bladder contractility index (BCI)	40.2 ± 25.1

**Table 2:** Parameters before and after the TUI-BN plus ultrasound-guided external sphincter BoNT-A injection.

	Preoperation	1 Month after Operation	*p* Value	3 Months after Operation	*p* Value
Voiding volume (VV) (mL)	127.8 ± 172.0	192.6 ± 99.0	0.019 *	189.3 ± 133.6	0.009 *
PVR urine amount (mL)	358.1 ± 192.3	118.3 ± 75.0	<0.001 *	76.7 ± 73.8	<0.001 *
VE (%)	24.6 ± 20.5	61.7 ± 17.6	<0.001 *	73.1 ± 23.7	<0.001 *
CIC (times/day)	3.4 ± 2.	1.3 ± 1.1	0.002 *	0.5 ± 1.1	0.002 *
AUA symptoms score	24.6 ± 5.3	12.3 ± 6.9	0.001 *	8.0 ± 5.9	<0.001 *
Storage symptoms	8.7 ± 2.7	7.9 ± 5.1	0.23	3.8 ± 3.1	0.001 *
Voiding symptoms	15.9 ± 3.9	2.8 ± 1.1	<0.001 *	4.2 ± 3.7	<0.001 *
QoL on AUA symptoms score	4.9 ± 1.1	4.9 ± 2.4	0.804	1.3 ± 0.9	0.001 *
PPBC	4.4 ± 1.0	2.1 ± 1.2	0.001 *	0.7 ± 0.9	<0.001 *

* means statistically significance (*p* < 0.05).

## Data Availability

The data presented in this study are available on request from the corresponding authors. The data are not publicly available due to the privacy protection policy.

## Supplementary Materials

The following supporting information can be downloaded at https://www.mdpi.com/article/10.3390/toxins16100441/s1; Table S1. Primary data of the 16 women with UAB symptoms and chronic urine retention.
